# Fretting and Fretting Corrosion Processes of Ti6Al4V Implant Alloy in Simulated Oral Cavity Environment

**DOI:** 10.3390/ma13071561

**Published:** 2020-03-28

**Authors:** Marcin Klekotka, Jan Ryszard Dąbrowski, Katarzyna Rećko

**Affiliations:** 1 Institute of Biomedical Engineering, Bialystok University of Technology, Wiejska 45C, 15-351 Bialystok, Poland; j.dabrowski@pb.edu.pl; 2 Faculty of Physics, University of Białystok, Ciołkowskiego 1L, 15-245 Białystok, Poland; k.recko@uwb.edu.pl

**Keywords:** fretting corrosion, implants, saliva, wear, biomaterials, titanium alloy

## Abstract

The paper presents the results of in vitro studies of fretting and fretting corrosion processes of Ti6Al4V implant alloy in the environment of natural saliva and self-made mucin-based artificial saliva solutions. The study was performed on a specially designed fretting pin-on-disc tester, which was combined with a set used for electrochemical research. The open circuit potential measurements and potentiodynamic method were used for corrosion tests. The worn surfaces were subjected to microscopic observations and an evaluation of wear. Results were interpreted using the dissipated energy and third-body approaches. The X-ray diffraction analysis showed that titanium oxides constitute over 80% of the friction products. Special attention was paid to the role of saliva and its substitutes, which in certain cases can lead to the intensification of fretting wear. On the basis of the received results, a new phenomenological model of fretting corrosion processes was proposed. This model involves the formation of an abrasive paste that is a combination of metal oxides and the organic components of saliva.

## 1. Introduction

Titanium and its alloys are materials that are particularly frequently used in alloplasty as well as in dental prosthetics and surgery. Titanium’s high corrosion resistance and good biocompatibility are due to the formation of a stable layer of oxides (TiO, Ti_2_O_3_, and TiO_2_) with a thickness ranging from 20 to 100 Å [1,2]. Titanium can be used in commercially pure form (Cp, Ti) as well as in alloy form. Alloy in bi-phase form (α and β) with the addition of aluminum and vanadium has been most commonly used for medical applications for many years [3]. Due to the toxicity of vanadium, which causes osteolysis, inflammatory states as well as carcinogenic changes [4], new alloy compositions (Ti-6Al-7Nb, Ti-6Al-(3–6)Nb-(1–6)Ta, Ti-5Al-2.5Fe, Ti-12Mo-5Zr-3Al) with better biocompatibility are being created. Additives of Mo, Ta, and Nb stabilize the β-phase and improve their fatigue strength, corrosion resistance, and elastic modulus, which should be similar to the elastic modulus of human bone [5,6]. However, due to their high production costs, their involvement in medical applications is limited [7,8]. Moreover, the majority of titanium alloys contain aluminum as an alloying agent, the ions of which damage nerve cells and block the bonding of proteins responsible for their regeneration. Long-term exposure to an elevated level of Al^3+^ ions may lead to neurological problems, dementia, and brain diseases [9,10].

A flaw of titanium implants is also their low wear resistance. They are many studies about the tribological properties of titanium alloys. Ti6Al4V is one of the most commonly used titanium alloys for biomedical device manufacturing [11]. Even nowadays, when there are a lot of new biomaterials, researchers still investigate the wear resistance of Ti6Al4V alloy, which has been in use since the 1950s and has been intensively investigated up to the present times [12,13]. Despite significant progress achieved in improving the quality of medical alloys, the price of a biofunctional material is often a decisive factor. It is known that the application of the identical materials in friction nodes of medical constructions is not recommended due to the elevated level of wear, particularly adhesive wear. However, in the case of parts of nominally immobile joints, it is possible to select identical materials, and this is done to limit the phenomenon of galvanic corrosion (e.g., some retention elements of dental prostheses) [14]. Hence, familiarity with the wear mechanisms of the most commonly applied biomaterials is expedient, particularly in the case of complex prosthetic and orthodontic systems exposed to the environment of saliva. In this case, fretting and corrosion processes are particularly dangerous, as they result in intensive destruction of joints in constructional components [15,16]. In prosthetics, fretting corrosion has been pointed out as one of the main failure mechanism. The parts most exposed to wear are: dental implant–abutment connections, attachment systems for mandibular overdenture (e.g., ball attachments), prosthetic bridges, and points of fixation of the implant with the mandible [17,18]. In the case of orthodontic appliances, arches and brackets, above all, are exposed to wear [19]. This causes a series of complications and significant deterioration of patients’ comfort in their healthcare. Fretting wear is usually abrasive–fatigue in nature, where microdisplacements lead to abrasion phenomena, and periodic vibrations influence the material’s fatigue processes. However, when friction is combined with an actively interacting environment, fretting corrosion occurs, intensifying material wear processes. It leads to excessive wear and loss in the performance of dental implants, especially their congruent components and pressing elements. The resulting slacks negatively affect the functioning of the entire implant structure. In practice, different wear mechanisms (abrasion, adhesion, and oxidation) typically co-exist, of which one assumes a dominant character [20,21].

Another problem in the analysis of fretting wear is accounting for the geometric conditions of the friction pair’s contact surface. Due to simple measurements and easy identification, most wear tests are performed with concentric contact in ball-on-disc systems, in which one central area of wear is present [22,23]. In many dental systems, two flat elements are often joined, and the contact between them is congruent. Then, analysis of fretting wear is rendered significantly more difficult due to the different shapes and sizes of surface damage, which are situated accordingly to the actual contact surface. Congruent contact also makes it difficult to remove wear products from the friction node, intensifying the secondary wear of the material and enlarging randomly distributed micro-areas of wear [24]. 

There are many studies of the destruction mechanisms of materials under fretting corrosion conditions [25,26,27]; however, due to the complexity of the phenomenon, it is not fully understood in the authors’ opinion, particularly from the perspective of assessment of the wear of metallic biomaterials in the environment of saliva and its substitutes. This environment has a varying, often invasive, nature. The substances present in saliva (bacteria, fungi, enzymes, food residue, proteins) can have a significant impact on the progression of tribological processes [28]. Mucins, glycoproteins ensuring the proper viscosity of saliva and lubrication of the dental apparatus, play a particularly significant role [29,30]. 

The tests described in this paper were carried out on our own self-made compositions of artificial saliva with beneficial lubricating properties. However, in some sliding conditions, when the amplitude of oscillation is small, lubricants may intensify the fretting wear of titanium dental alloys and slow down the migration of wear debris outside of the friction area. The main aim of this work is to present a suggestion for an explanation of the fretting wear mechanism for Ti-Al-V titanium alloy in the environment of saliva and its substitutes. In the majority of papers dealing with the assessment of friction and wear of prosthetic and orthodontic materials, the measurements are carried out in the ball-on-disc system. Our studies were performed for the flat-on-flat system. The contact geometry presented in this model is more difficult to analyze, but in our opinion, it corresponds better with multi-component dental implant systems (e.g., abutment screws). Moreover, the analysis of abrasive wear products may facilitate the assessment of the toxicity of a biomaterial. 

## 2. Materials and Methods

**Figure 1:**
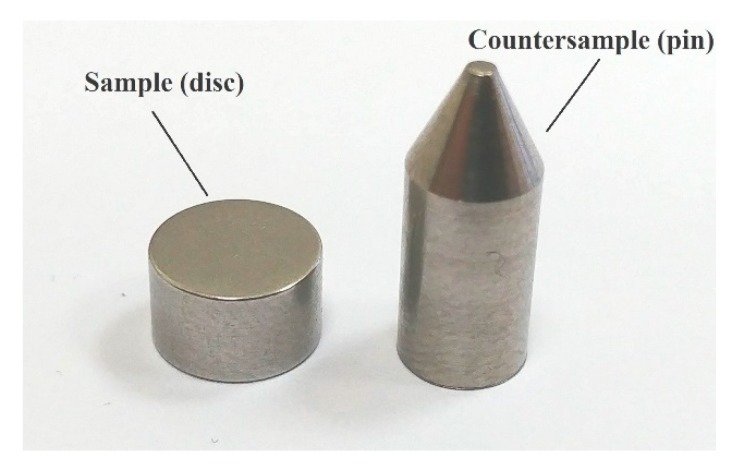
Image of the samples’ geometry.

Tests were performed using Ti6Al4V implant alloy. Samples in the shape of discs with a diameter of 8 mm and thickness of 5 mm were made from metal rods and then subjected to mechanical grinding. The magnetic grinder (Jotes, Łódź, Poland) used for this purpose allows obtaining the roughness of the samples Ra = 0.4 μm. In response to the high repeatable values of the roughness, the authors decided to not polish the surfaces of the samples. Moreover, the higher roughness of the implant increased the active surface area and improved cells’ adhesion, which affects osseointegration. For that reason, most of the commercial dental implants have a microroughened surface [31]. Countersamples in the shape of a truncated cone were made of rods with a thickness of 6 mm, and the diameter of the surface in contact with the sample was reduced to 1.3 ± 0.1 mm. Samples were rinsed with water and cleaned in ethanol for 10 minutes in an ultrasound bath. The pin and disc were made of the same material. Its chemical composition is presented in Table 1. The geometry of the samples is shown in Figure 1.

Saliva for the tests was collected from a healthy, 29-year-old man according to the previously developed methodology [32]. Artificial saliva compositions were made based on phosphate-buffered saline (PBS) with the following chemical composition: NaCl (6.72 g/L), Na_2_HPO_4_ (1.27 g/L), and KH_2_PO_4_ (1.11 g/L). Substitutes were selected based on analysis of the literature and the authors’ original studies [33]. Table 2 presents the formulas used in tests, containing additions of mucin III and xanthan gum.

The selection of mucin was linked to the beneficial lubricating characteristics of this substance. Mucins are large high-molecular-weight glycoproteins, which are present in natural saliva. Their oligomeric structure and elongated form in combination with a hydrophilic sugar coat provides the saliva proper viscosity and reduces the friction forces [34]. Xanthan gum is a water-soluble extracellular polysaccharide that is widely used as a stabilizer and emulsifier in the food, pharmaceutical, and cosmetics industries. It is also a commonly used ingredient in many formulas intended for application in the oral cavity. Xanthan gum made it possible to correct the rheological properties of the developed formulas with respect to natural saliva. All formulas had a pH similar to that of natural saliva (6.8–7.1). A more detailed physicochemical and rheological analysis of saliva and it substitutes was presented in our other publications [35,36].

**Figure 2:**
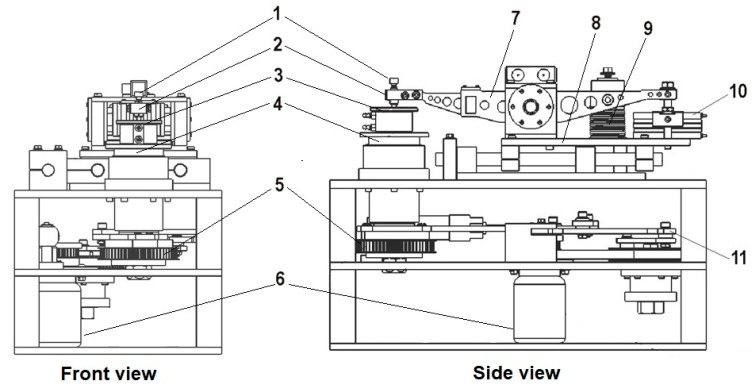
Schematics of the fretting tester: 1—screw, 2—countersample holder, 3—sample holder, 4—rotating table, 5—wheel driving the rotating table, 6—driving motor, 7—lever, 8—slidable table, 9—loading spring, 10—pneumatic actuator, 11—lever system.

Fretting and fretting corrosion processes were carried out using a fretting tester (Białystok University of Technology, Białystok, Poland) designed and built at the Białystok University of Technology. The tribometer with a mechanical system affects oscillating movement and ensured flat contact between the moving sample (disc) and immobile countersample (pin). The spring and pneumatic actuator allows gaining a proper load. The lever system is responsible for a sample displacement amplitude regulation [37]. A diagram of the device is shown in Figure 2.

Fretting tests were performed under dry sliding conditions and in the presence of saliva and its substitutes. Fretting was realized at room temperature (21 ± 1 °C) for one hour (2880 cycles) with amplitude of 100 µm, at 0.8 Hz frequency, and mean unit loads of 5, 15, and 30 MPa. The movement frequency is within the range of professional chew testers [38]. Unit loads were selected to be as similar as possible to actual loads occurring in the human oral cavity [39]. According to Amonton’s law, the friction coefficient is the ratio of tangential force to the normal force. The tangential forces were estimated as the average of the absolute values of the minimum and maximum forces obtained by strain gauges in the final cycles of fretting. Friction coefficients for the kinematic pairs were calculated. After rubbing, the wear traces were assessed under a microscope. Besides volumetric wear measurement, analysis of the energy dissipated in a single friction cycle was conducted. The energy is dissipated when two surfaces rub together. A certain amount of work would need to be done by the force to sustain the sliding motion. Frictional work can be converted into heat, which suggests it is an energy process [40]. It should be noted that dissipated energy is a useful indicator for evaluating fretting damage [41].

**Figure 3:**
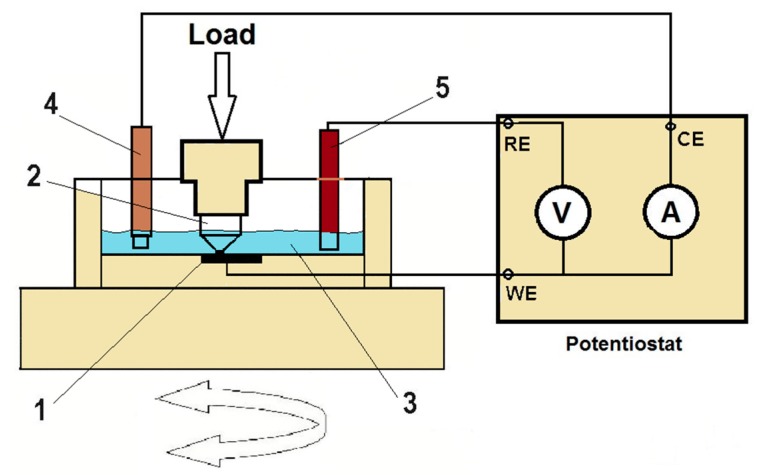
Friction pair and corrosion measurement system: 1—sample, 2—countersample, 3—electrolyte, 4—auxiliary electrode, 5—reference electrode.

To perform corrosion tests under friction conditions, a PGP201 potentiostat (Radiometer Analytical, Lyon, France) was connected to the fretting tester. Electrochemical measurements were performed in a tri-electrode configuration. The reference electrode was a saturated calomel (Hg/Hg_2_Cl_2_/Cl^−^) electrode REF421 (Radiometer Analytical, Loveland, CO, USA), the potential of which relative to that of a normal hydrogen electrode was +0.244 V. The auxiliary electrode was an XM140 platinum electrode (Radiometer Analytical, Loveland, CO, USA), with an area of 8 × 8 mm, and the working electrode was a kinematic pair made of titanium alloy. Samples were placed in a specially designed grip ensuring that only the face surface was in contact with the electrolyte, while simultaneously enabling the connection of the potentiostat to the sample. A diagram of the friction pair and corrosion measurement system is shown in Figure 3.

Electrochemical processes under sliding conditions were registered at room temperature with an amplitude of 100 µm at 0.8 Hz frequency and under a unit load of 15 MPa. During tests, two types of electrochemical measurements were carried out: open-circuit potential (OCP) and potentiodynamic tests. OCP tests were performed in three stages. Samples were immersed in electrolyte, and changes of potential were registered as a function of time. The first stage covered measurements under static conditions, the second covered measurements under fretting conditions under the set load, and the last covered measurements after the end of sliding. To enable the relative stabilization of the measured values, each of the stages lasted one hour. Corrosion investigations using the potentiodynamic method under fretting conditions were performed for three types of independent tests:

- I—reference test, a typical pitting corrosion resistance test performed based on standard [42].
- II—corrosion resistance tests of materials with surface damaged as a result of fretting.
- III—changes of electrochemical parameters were registered over the course of fretting.

After samples were conditioned in electrolyte for one hour, changes of their open circuit potential were registered under static conditions (before and after sliding) and dynamic conditions (during sliding). The polarization process under fretting conditions started from potential E_start_ = E_OCP_ − 100 mV. Potential change progressed in the direction of the anode at a rate of 3 mV/s after reaching the maximum value of the measuring range +2000 mV (relative to Hg/Hg_2_Cl_2_/Cl^−^).

For statistical purposes, every tribological and electrochemical test was repeated three times. It allowed calculating the mean values and estimating the standard deviations of the obtained results.

Microscopic analysis made it possible to assess the fretting wear of samples. The wear assessment method uses a dense mesh of profiles on the surface morphology. According to this approach, it is possible to differentiate between decrements of material (pits below the material’s surface layer) and an excess of material (areas above the material’s surface layer). The details of that method were described in our previous publication [37]. Surface observations were conducted using confocal laser scaning microscope (CLSM) Lext OLS4000 (Olympus, Tokyo, Japan), Phenom XL scanning electron microscope (SEM) integrated with an energy-dispersive detector (Phenom-World, Eindhoven, Netherlands), and transmission electron microscope (TEM) Tecnai G2 X-TWIN (FEI, Hillsboro, OR, USA).

For CLSM analysis, samples were air-dried at temperature 21 ± 1 °C for 24 h. In case of SEM observations, to provide better electron conductivity, samples were rinsed with a small amount of distilled water before drying. TEM investigations were performed only for samples after dry sliding. Wear debris were collected with a brush to small test tubes and dispersed in 96% ethanol with the use of an ultrasound bath. Then, a drop of ethanol with debris was placed on a Cu grid (300 mesh) and left until the solvent evaporated.

The phase composition of the top layer modified in the friction process was determined by means of X-ray diffraction (XRD). Samples tested in dry sliding conditions were subjected to XRD analysis immediately after the sliding ended. The total area of the discs’ surfaces with accumulated wear debris was investigated. The room temperature XRD measurements were performed using an Empyrean diffractometer (Malvern Panalytical B.V., Almelo, Netherlands) equipped with an X-ray tube with a Cu anode. The diffractometer worked in Bragg–Brentano geometry, and the scattered intensity was recorded using a PixCel1D strip detector within a 2θ range from 5° to 50°.

## 3. Results and Discussion

**Figure 4:**
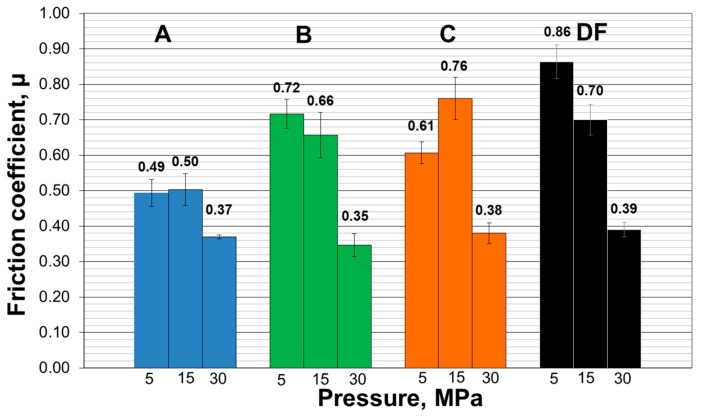
Impact of human saliva and its substitutes on friction coefficient values under stable conditions: A—human saliva, B—mucin III in PBS, C—mucin III + xanthan gum in PBS, DF—dry friction.

Test results indicate that in the majority of the analyzed cases, the coefficients of friction decrease as unit loads increase. It may be related to the fact that under high pressure, wear products are crushed and their migration beyond the friction zone is limited. A lot of the particles remain in the friction zone. These particles reduce the adhesion between surfaces and thus reduce the coefficient of friction. The results of the friction coefficient measurements are shown in Figure 4.

**Figure 5:**
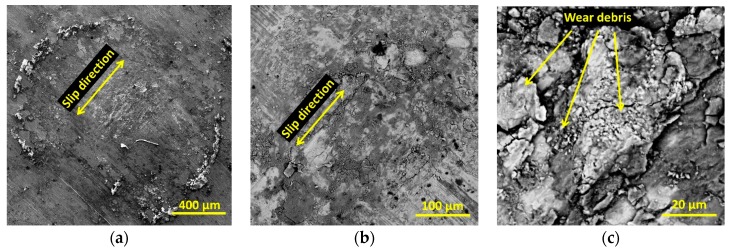
SEM images of surface morphology after a fretting test in an artificial saliva environment (solution B, 15 MPa). (**a**) 400 μm; (**b**) 100 μm; (**c**) 20 μm.

In case of smaller unit loads, the wear products are easier removed from the friction zone. Uncovered metallic surfaces are susceptible to adhesive tacking and intensive oxidation processes, which leads to the growth of friction coefficients. Example SEM images of the friction zone morphology are shown in Figure 5.

A significant influence of saliva and its substitutes, changing values of friction coefficients, was also observed. The boundary films formed on the sliding surface reduce the values of friction coefficients and exhibit greater effectiveness at lower unit loads (5 MPa) in comparison to dry friction. Natural saliva seems to be an optimal environment, with beneficial lubricating properties. However, at high unit loads (30 MPa), the friction coefficients are similar for both conditions, under dry friction and in the presence of lubricants. This suggests that at the high pressures, the friction coefficient is more dependent on the wear conditions e.g., sliding time, type of material, or surface roughness than on the type of used lubricant. 

The dependencies seen here can be explained by the formation of adsorptive boundary films on sliding surfaces, which are capable of reducing the coefficient of friction [29,43]. These films are built of organic ingredients of saliva and its substitutes, mainly glycoproteins, including mucins. However, their adsorptive capacity is relatively small. At high loads, they are removed from the friction surface, and values of friction coefficients become more similar to those obtained under dry friction conditions.

**Figure 6:**
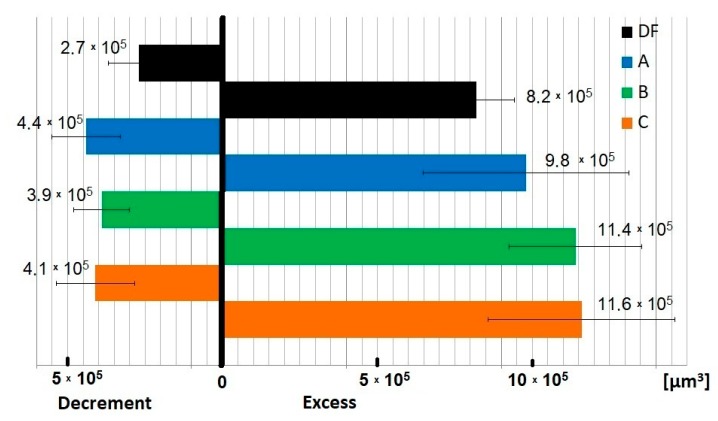
Results of fretting wear measurement, accounting for volumes of material decrement and excess (p = 30 MPa).

It should also be emphasized that demonstrating unambiguous and repeatable wear values of the tested samples was problematic due to the wear mechanisms specific to fretting, particularly regarding difficulties with removing formed products from the friction zone. Some of these products remained in the contact zone, and procedures for their removal did not bring the expected results. In relation to this, the quantitative assessment of fretting wear that was conducted should be considered an estimate, indicating specific tendencies. According to the developed methodology [37], wear products moved outside of the friction zone were assessed as “excess”, and wear in the contact area was assessed as “decrement”. As an example, wear test results are given in Figure 6.

**Figure 7:**
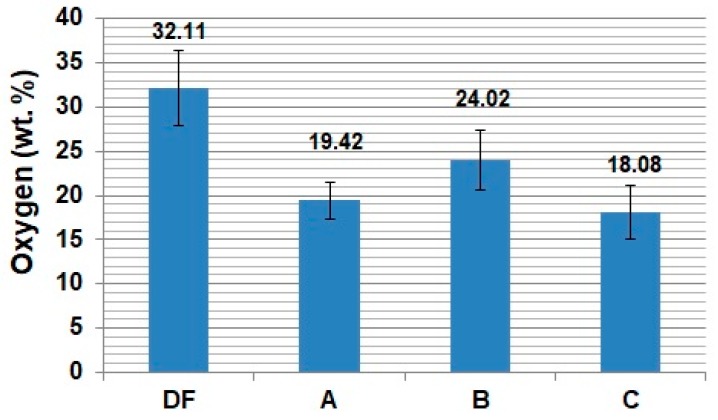
Oxygen content in wear products (p = 30 MPa).

The obtained test results indicate that in the case of the uncovered metallic surface of titanium alloys, which is characterized by high chemical reactivity [44], saliva and its substitutes may cause intensification of third-body abrasive wear. This occurs especially at higher unit pressures, where the removal of wear products outside the friction zone is difficult. Simultaneously, an increase of the oxygen presence in wear debris was observed. Results of the oxygen content measurement are presented in Figure 7.

It seems that the oxides formed in the friction zone, mixed with ingredients of the tested lubricants, may form boundary films in the form of a unique “abrasive paste”. This paste, only partially removed from the edge of the friction zone, constituted a binder for the wear products that formed. The elevated amount of particles formed in the friction zone could have intensified secondary wear processes. Some of these products remained on contact surfaces even after cleaning the sample surfaces, creating clusters. This was observed in the form of a relatively large amount of excess (Figure 6).

**Figure 8:**
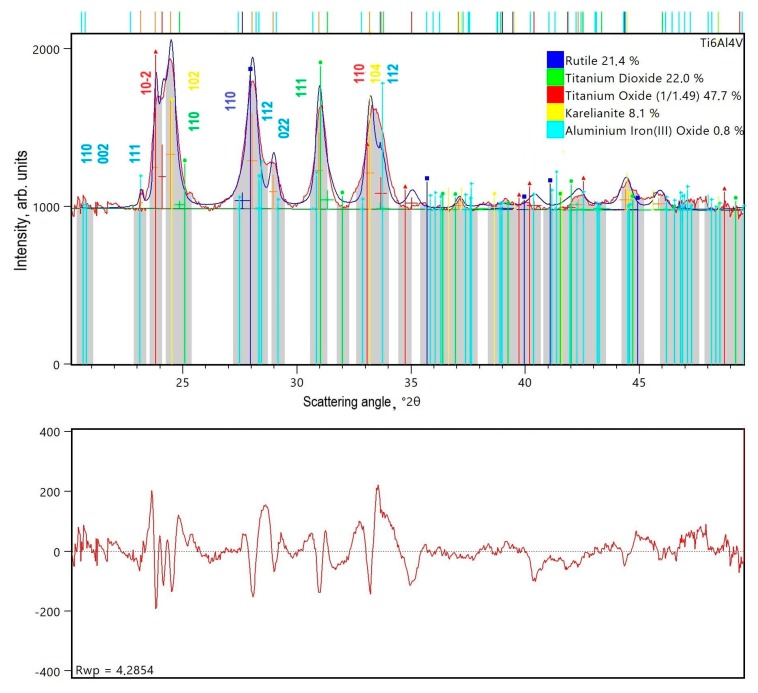
Room temperature X-ray powder diagrams experimental (red solid line), calculated (blue solid line), and the residual one (panel below) for the friction products of Ti6Al4V alloy.

XRD phase analysis of titanium alloy wear products indicated the formation of oxides, primarily titanium (IV) and vanadium (III), and traces of a triple-component compound based on aluminum (III) and iron (III). The compositional homogeneity of the sample and then the order of each structure were analyzed by means of the phase analysis software package HighScore [45]. Due to peak broadening, the two most important treatments are background determination and peak search. Noticeably, a proper background determination is very important for correct phase analysis, which is using the measured net profile data, while peak search is required during profile fitting and indexing. The (hkl)-values are the direct results of Rietveld refinement of the data. The largest contributions of an assembled X-ray profile belong to various forms and structures of metal tri- and dioxides. Titanium oxides constitute over 80% of the friction products. The X-ray diagram shows 47.7% contribution of the hexagonal phase Ti_2_O_2.98_. The crystal symmetry of titanium (III) oxide is described according to the card ICSD No. 98-002-4292 as an R-3c space group (No. 167) with the following unit cell parameters: a = b = 516 pm, c = 1360 pm. Two similarly numerous friction products (slightly more than 20% each) are tetragonal rutile (ICSD No. 98-007-4532; P42/mnm (sg. 136)) with 451 pm and 303 pm of cell parameters and orthorhombic titanium dioxide (ICSD No. 98-018-9320; Pbcn (sg. 60)) with unit cell parameters of 459 pm, 559 pm, and 494 pm. A small contribution to the diffraction pattern brings V_2_O_3_ (Karelianite) that crystallizes in the hexagonal symmetry (ICSD No. 98-020-1109; R-3c, sg. 167 with unit cell parameters of a = b = 490 pm, c = 1400 pm). The traces of orthorhombic AlFeO_3_ (ICSD No. 98-020-3203; Pna21 (sg. 33) with 498 pm, 855 pm and 924 pm) are also observed. The appropriate agreement factor R_wp_—the weighted profile R-factor—was calculated according to the Rietveld algorithm [46]. For better readability, only well separated and the strongest Bragg reflections were indexed (Figure 8).

**Figure 9:**
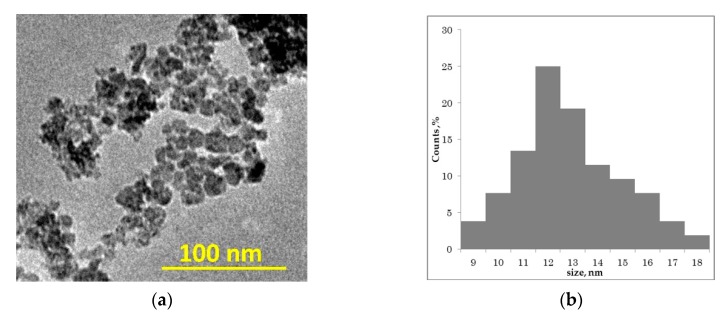
(**a**) TEM image of fretting wear products (dry friction, p = 15 MPa); (**b**) size distribution of nanoparticles.

The results of the diffraction data refinement are presented in Figure 8, while a photograph of exemplary wear products, taken using the TEM technique, is shown in Figure 9.

In spite of the high number of counts accumulated in peaks, the resolution functions of broadened lines are coupled with the microstrain coefficient and size distribution effect. The mean size of wear products was 27 nm for Ti_2_O_2.98_ crystallites, 7.8 nm for rutile crystallites, and 15 nm for orthorhombic titanium dioxide crystallites.

An analysis of the TEM image presented in Figure 9 clearly shows that obtained wear debris are in nanoparticles form. They are inhomogenous in size, strongly agglomerated, and the size distribution (Figure 9b) shows the wide range of their size. The mean size of nanoparticles was calculated to be 13 ± 2 nm. The fact of their heterogeneity and agglomerated form is connected with their preparation. In the dry friction process, there are no surface stabilizers that could separate nanoparticles and arrange them in a homogenous shape. One can see that the nanoparticles size calculated by TEM is different from the XRD results. Its origin is connected with the fact that on TEM images, different phases cannot be distinguished; therefore, only the mean size of seen objects can be analyzed.

**Figure 10:**
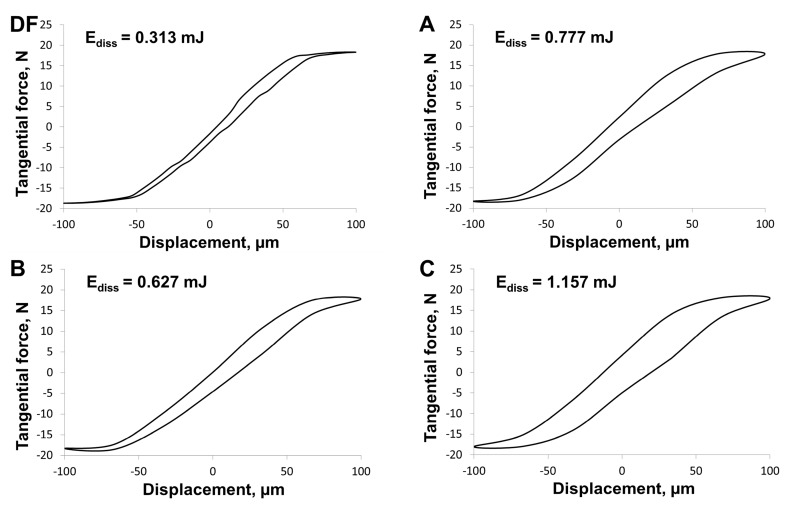
Energy dissipated during the single cycle of fretting (p = 30 MPa).

It seems to be of interest to analyze charts illustrating the dependency of tangential force with respect to displacement under fretting conditions. The evolutions of these curves provide information about the character of these displacements. The stick regime, partial slip regime, and gross slip regime are distinguished [47]. Curves for single fretting cycle, registered in the 60^th^ minute of sliding under dry friction (DF) conditions and in the environment of saliva (A) and its substitutes (B, C) are shown in Figure 10.

The character of obtained curves indicated partial contact with micro-slips. According to the assumption of Fouvry et al. [48], the wear of materials under fretting conditions is dependent on the energy dissipated (E_diss_) in every friction cycle. The amount of energy can be identified with the size of the hysteresis loop. As its area grows, the energy dissipated increases along with the degree of material wear. This assumption is best confirmed in the case of friction throughout the entire contact area. The tests described in this article confirm this dependency (Figure 6), even in the case where slips occur on part of the contact area. Dissipated energy was the lowest in dry friction (E_diss_ = 313 μJ), which can be associated with an easier removal of wear products outside the friction zone. In the case of friction under lubrication conditions, the hysteresis loops have greater areas than under dry friction conditions, which translates to greater wear of the biomaterial. It can be noticed that hysteresis loops are similar for substitute B (E_diss_ = 627 µJ) and natural saliva (E_diss_ = 777 µJ), which means that the degree of fretting destruction of prosthetic elements made of Ti6Al4V alloy should be comparable, both in healthy patients and using saliva substitute with the addition of mucins. A higher value of dissipated energy for composition C (E_diss_ = 1157 µJ) may be caused by the addition of xanthan gum, affecting the rheological properties of the lubricant and thus affecting the mobility of the wear products. From the obtained results, it can be concluded that the presence of a lubricant can intensify the tribological wear of titanium alloy under fretting conditions.

**Figure 11:**
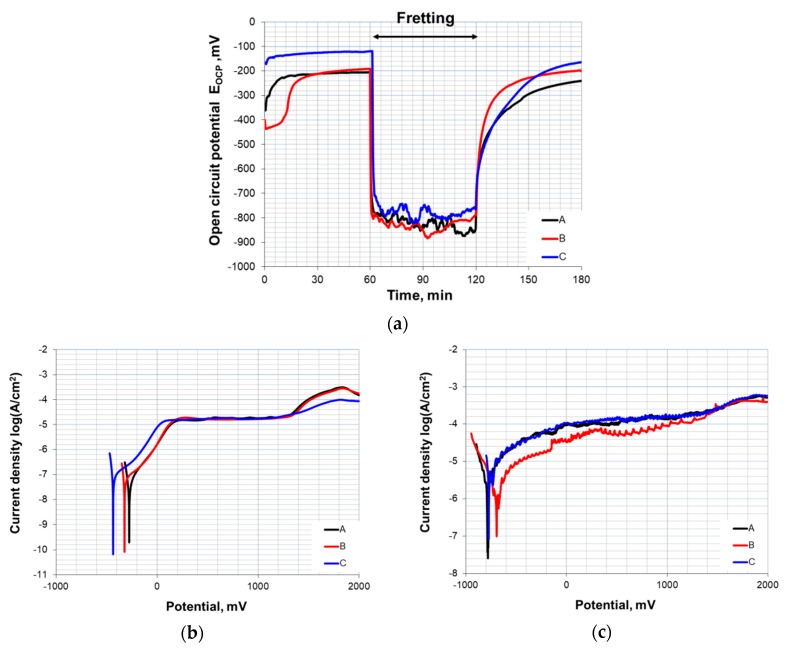
Electrochemical results of fretting corrosion tests: (**a**) Open circuit potential curves, (**b**) anodic polarization in static conditions, and (**c**) anodic polarization in dynamic conditions.

The results of electrochemical tests under fretting conditions for Ti6Al4V alloy are presented in Figure 11.

In open circuit potential tests (Figure 11a), three stages can be distinguished. In the first hour, the growth and stabilization of potential values occurred. This indicates the processes of hydrating and modifying a passive layer on the alloy’s surface, improving samples’ corrosion resistance. Next, the friction process was initiated. Open circuit potential values dropped substantially; however, due to the reactivity of the tested alloy and the continuous destruction and renewal of the oxide layer, potential changed within the range of ±50 mV. After fretting was stopped, open circuit potential began to rise; however, the time required for it to stabilize varied and was largely dependent on the type of environment. The mean values of potential differences between static and dynamic (fretting) conditions are presented in Table 3.

The drop in open circuit potential was similar for all environments; however, the results indicate that the addition of xanthan gum to formula C had an adverse impact on the alloy’s repassivation capacity. The tendency of accelerated repassivation is clearly visible as the amount of organic compounds in saliva substitutes is limited. The results of corrosion and fretting corrosion resistance tests obtained by the potentiodynamic method are presented in Table 4, Figure 11b,c.

**Figure 12:**
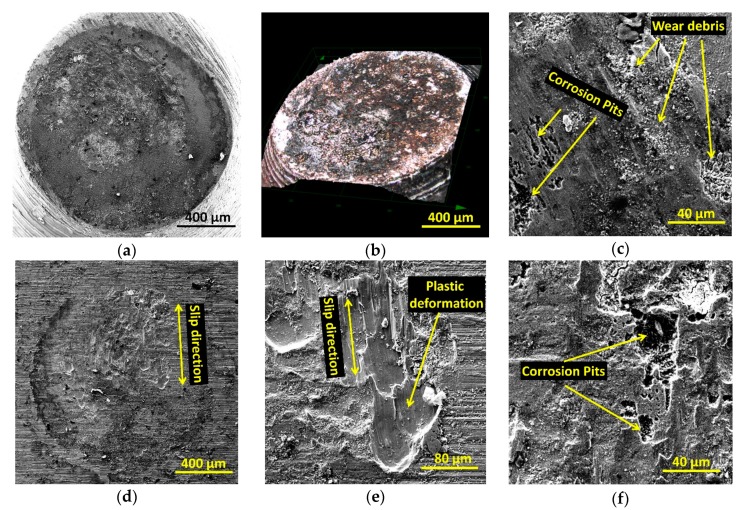
Surfaces of samples after fretting corrosion tests: (**a**–**c**)—pin, (**d**–**f**)—disc.

Particular attention was paid to the corrosion potential (E_cor_) and polarization resistance (R_P_). During corrosion tests under static conditions (without fretting), samples show high values of polarization resistance, particularly in natural saliva, which indicates the presence of a persistent passive film that effectively protected the metal against corrosion. For all environments, the passive range was up to approximately 1.4 V. Above this value, the transpassivity occurs (due to the oxidation of water [44]). Mechanical damage of the surface layer reduced the corrosion resistance of each of the tested samples. This was particularly visible during the registration of electrochemical changes over the course of friction. The evolution of potentiodynamic curves indicates strong irregularities, especially under passive range (approximately from 0 to 1.4 V). These irregularities are probably related to the crushing and smearing of the particles left by the mechanical abrasion of the passive oxide layer. The depolarization of an anodic reaction may also be the result of electron transfer and the dissolution of this layer. Obtained results indicate that the corrosion potentials of samples in the environment of artificial saliva, B, were shifted in the positive direction with respect to the other formulas. Moreover, they were also characterized by greater polarization resistance, which could be indicative of this environment’s favorable anti-corrosion properties. However, it should be noted that the layer formed as a result of repassivation undergoes mechanical degradation more easily, thus revealing the metal’s active surface and intensifying its wear. Analysis of co-existing electrochemical and mechanical phenomena from the perspective of a given material’s operational lifetime is a complex task, and the interpretation of obtained test results is not always simple and unambiguous. Microscopic observations of the tested elements surfaces are often a helpful part of research. Obtained results show that fretting corrosion processes lead to plastic deformations and the forming of corrosion pits at the fretted areas. In addition, a large amount of small wear debris was noticed. Examples of images obtained during fretting corrosion tests are presented in Figure 12.

**Figure 13:**
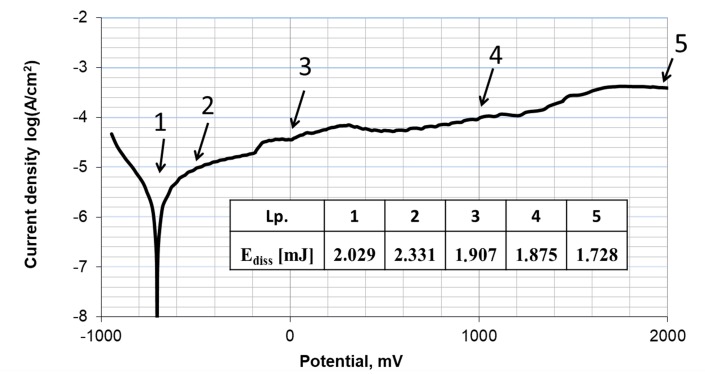
Energy dissipation during fretting corrosion (p = 15 MPa) in artificial saliva (B).

In this work, attention was also paid to the energetic aspect of potentiodynamic fretting corrosion tests. As the potential changed, dissipated energy was registered in individual friction cycles, and the obtained results are presented in Figure 13.

During friction, dissipated energy was the greatest at the initial stage of the test, which corresponded to the area of active solubilization of the metal (1,2). For passivation potentials (3,4), the energy decreased slightly, and it reached its lowest values within the transpassive range (5). Observed energy dependencies may be linked to electrochemical processes occurring on the surface of the tested alloy, affecting the operational lifetime of the surface layer. On the other hand, obtained results may indicate mechanical “grinding in” of contact surfaces, which also leads to a reduction of dissipated energy. However, this issue requires more in-depth analysis and will be continued in future research.

**Figure 14:**
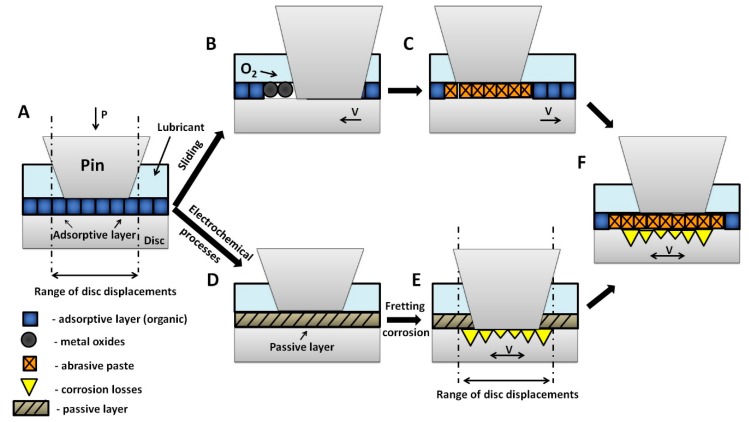
Phenomenological model of fretting corrosion processes: (**A**)—destruction of adsorptive layer, chemical oxidation, (**B**)—oxides accumulation, (**C**)—abrasive paste formation, (**D**)—passive layer, (**E**)—fretting corrosion (intensification of corrosion processes), (**F**)—tribological and corrosive wear.

The main purpose of this research is a better understanding of the wear mechanisms and proposal of a phenomenological model (Figure 13) of the fretting wear of metallic biomaterials on the example of Ti6Al4V alloy. The proposed model is shown in Figure 14.

In a natural oral cavity environment, the saliva covers teeth, periodontium, tongue, and mucous membrane with a thin 70–100 μm film. On the hard palate, the film thickness is below 10 μm [49]. The model assumes that under static conditions, the alloy’s surface is covered with a layer of adsorbed organic ingredients, which make up compounds in saliva and its substitutes (Figure 14A). Its chemical composition is strictly dependent on the composition of saliva. The adsorptive layer mainly consists of polysaccharides and glycoproteins (including mucins), which may have a beneficial influence on friction conditions due to their structure and viscoelastic properties [50]. It should be noted that the adsorptive capacity of organic film is relatively small. The removal of this layer uncovers the metal’s active surface. This leads to an increase in the adhesive wear and oxidation processes (Figure 14B). The progression of wear depends on friction conditions and the reactivity of materials in the friction pair. Formed oxides usually cause secondary wear processes. The presence of organic lubricants may hinder oxygen access to uncovered metallic surfaces while at the same time impeding the migration of wear particles outside of the friction area. The formed “abrasive paste” containing hard metal oxide particles suspended in lubricant intensifies the wear processes (Figure 14C). In fretting wear, there is synergy between friction and corrosion processes (Figure 14F). Passive films on the alloy’s surface (Figure 14D) reduce the intensity of electrochemical processes. Titanium alloys are particularly susceptible to passivation processes. However, as a result of rubbing and uncovering of the metal’s active surface, metal solubilization (corrosion) may occur (Figure 14E). It is also worth mentioning that the abrasive paste formed during fretting may reduce the access of oxygen to the contact area and slow down the repassivation of metal. This low oxygen region is an anode, where the corrosion processes occur more intensively. The area beyond the friction zone has a higher oxygen concentration, which makes it a cathode [35].

## 4. Conclusions

The realized study allows understanding biomaterials’ wear mechanisms better, especially in a simulated oral cavity environment. This knowledge is essential for the proper selection of materials’ prosthetic and orthodontic applications. The following conclusions can be drawn:

- Due to the high surface reactivity and capacity for rapid formation of a passive layer, titanium and its alloys are characterized by high corrosion resistance. However, this pertains to static conditions.
- The long-term, systematic abrasion and oxidation of the surface layer results in the formation of a large amount of wear products and in a significant reduction of the materials’ corrosion resistance.
- It has been noticed that at the high-pressure sliding (30 MPa), the coefficient of friction slightly depends on the kind of artificial saliva.
- In certain cases, lubricants in the form of saliva and its substitutes may intensify the fretting wear of parts in prosthetic and orthodontic applications made from Ti6Al4V titanium alloy. An attempt to explain this phenomenon is described in the proposed fretting wear model.
- The organic ingredients present in saliva and its substitutes reduce titanium alloy’s oxidation capacity. In the case of tribochemical processes, this is a beneficial phenomenon and may lead to a limitation in the amount of wear products.
- On the other hand, the presence of lubricants in sliding contact fostered the formation of abrasive paste and made it difficult to remove from the wear zone.
- The composition of phosphate-buffered saline and mucins (solution B) can be used as a model solution for studies on properties of artificial saliva. It can be modified according to required properties by various additives, i.e., xanthan gum, guar gum, metal nanoparticles, proteins, and others.

This work also directs attention to the energetic aspect of tribological processes under fretting and fretting corrosion conditions. However, the complexity of the correlation between wear and corrosion means that this problem is still current and requires further research.

## Figures and Tables

**Table 1:** Chemical composition of Ti6Al4V alloy (maximum content as a wt %).

Ti	Al	V	Fe	O	C	N	H
remainder	5.500	3.500	<0.300	<0.200	<0.080	<0.050	<0.0015

**Table 2:** Saliva and its substitutes. PBS: phosphate-buffered saline.

Lubricant	Composition
A	Human saliva
B	Mucin type III (2 wt %) in PBS
C	Mucin type III (2 wt %) + xanthan gum (0.35 wt %) in PBS

**Table 3:** Open-circuit potential (OCP) decrease caused by mechanical depassivation due to fretting.

Saliva	Natural (A)	Artificial (B)	Artificial (C)
ΔE_OCP_ [mV]	617 ± 48	638 ± 42	649 ± 51

**Table 4:** Results of corrosion and fretting corrosion tests.

Saliva	Test *	E_cor_ [mV]	R_p_ [kΩ⋅cm^2^]
Natural (A)	I	−273 ± 11	456.64 ± 5.37
	II	−310 ± 12	54.43 ± 3.67
	III	−783 ± 30	0.27 ± 0.11
Artificial (B)	I	−267 ± 6	266.29 ± 0.78
	II	−324 ± 15	76.18 ± 2.19
	III	−701 ± 22	16.51 ± 0.92
Artificial (C)	I	−436 ± 23	209.12 ± 0.99
	II	−205 ± 24	38.87 ± 1.22
	III	−772 ± 38	1.95 ± 0.09

* I—reference trial (static conditions); II—corrosion test after fretting; III—corrosion test during fretting (dynamic conditions).

## References

[1] Prasad S., Ehrensberger M., Gibson M.P., Kim H., Monaco E.A. (2015). Biomaterial properties of titanium in dentistry. J. Oral Biosci..

[2] Cordeiro J.M., Beline T., Ribeiro A.L.R., Rangel E.C., da Cruz N.C., Landers R., Faverani L.P., Vaz L.G., Fais L.M.G., Vicente F.B. (2017). Development of binary and ternary titanium alloys for dental implants. Dent. Mater..

[3] Shah F.A., Trobos M., Thomsen P., Palmquist A. (2016). Commercially pure titanium (cp-Ti) versus titanium alloy (Ti6Al4V) materials as bone anchored implants—Is one truly better than the other?. Mater. Sci. Eng. C.

[4] Ress N.B., Chou B.J., Renne R.A., Dill J.A., Miller R.A., Roycroft J.H., Hailey J.R., Haseman J.K., Bucher J.R. (2003). Carcinogenicity of inhaled vanadium pentoxide in F344/N rats and B6C3F1mice. Toxicol. Sci..

[5] Hacisalihoglu I., Samancioglu A., Yildiz F., Purcek G., Alsaran A. (2015). Tribocorrosion properties of different type titanium alloys in simulated body fluid. Wear.

[6] Diomidis N., Mischler S., More N.S., Roy M., Paul S.N. (2011). Fretting-corrosion behavior of B titanium alloys in simulated synovial fluid. Wear.

[7] Bolzoni L., Ruiz-Navas E.M., Gordo E. (2017). Evaluation of the mechanical properties of powder metallurgy Ti-6Al-7Nb alloy. J. Mech. Behav. Biomed. Mater..

[8] Siqueira R.P., Sandim H.R.Z., Hayama A.O.F., Henriques V.A.R. (2009). Microstructural evolution during sintering of the blended elemental Ti-5Al-2.5Fe alloy. J. Alloys Compd..

[9] Mirza A., King A., Troakes C., Exley C. (2017). Aluminium in brain tissue in familial Alzheimer’s disease. J. Trace Elem. Med. Biol..

[10] Avila J. (2018). Microtubule Proteins.

[11] Wang G., Dargusch M., Doan N. Material Perspectives of the Dental Implants: A Review. Proceedings of the 6th International Conference on the Development of Biomedical Engineering in Vietnam.

[12] Buciumeanu M., Bagheri A., Shamsaei N., Thompson S.M., Silva F.S., Henriques B. (2018). Tribocorrosion behavior of additive manufactured Ti-6Al-4V biomedical alloy. Tribol. Int..

[13] Philip J.T., Mathew J., Kuriachen B. (2019). Tribology of Ti6Al4V: A review. Friction.

[14] Byrne G. (2014). Fundamentals of Implant Dentistry.

[15] Zhou Z.-R., Yu H.-Y., Zheng J., Qian L.-M., Yan Y. (2013). Dental Biotribology.

[16] Sivakumar B., Kumar S., Sankara Narayanan T.S.N. (2011). Fretting corrosion behaviour of Ti-6Al-4V alloy in artificial saliva containing varying concentrations of fluoride ions. Wear.

[17] Yu H.Y., Gao S.S., Cai Z.B., Quan H.X., Zhu M.H. (2009). Dual-motion fretting behavior of mandibular cortical bone against pure titanium. Tribol. Int..

[18] Corne P., March P.D., Cleymand F., Geringer J. (2019). Journal of the Mechanical Behavior of Biomedical Materials Fretting-corrosion behavior on dental implant connection in human saliva. J. Mech. Behav. Biomed. Mater..

[19] Rapiejko C., Fouvry S., Grosgogeat B., Wendler B. (2009). A representative ex-situ fretting wear investigation of orthodontic arch-wire/bracket contacts. Wear.

[20] Geringer J., Forest B., Combrade P. (2005). Fretting-corrosion of materials used as orthopaedic implants. Wear.

[21] Zhu M.H., Yu H.Y., Zhou Z.R. (2006). Radial fretting behaviours of dental ceramics. Tribol. Int..

[22] Zhu M., Zhou Z. (2001). An experimental study on radial fretting behaviour. Tribol. Int..

[23] Zhu M.H., Zhou Z.R. (2011). On the mechanisms of various fretting wear modes. Tribol. Int..

[24] Swaminathan V., Gilbert J.L. (2012). Fretting corrosion of CoCrMo and Ti6Al4V interfaces. Biomaterials.

[25] Geringer J., Pellier J., Taylor M.L., MacDonald D.D. (2013). Fretting corrosion with proteins: The role of organic coating on the synergistic mechanisms. Thin Solid Film..

[26] Sato Y., Iwabuchi A., Uchidate M., Yashiro H. (2015). Dynamic corrosion properties of impact–fretting wear in high-temperature pure water. Wear.

[27] Tritschler B., Forest B., Rieu J. (1999). Fretting corrosion of materials for orthopaedic implants: A study of a metal/polymer contact in an artificial physiological medium. Tribol. Int..

[28] Levine M.J. (1993). Development of artificial salivas. Crit. Rev. Oral Biol. Med..

[29] Bongaerts J.H.H., Rossetti D., Stokes J.R. (2007). The lubricating properties of human whole saliva. Tribol. Lett..

[30] Pailler-Mattei C., Vargiolu R., Tupin S., Zahouani H. (2015). Ex vivo approach to studying bio-adhesive and tribological properties of artificial salivas for oral dryness (xerostomia). Wear.

[31] Gaviria L., Salcido J.P., Guda T., Ong J.L. (2014). Current trends in dental implants. Korean Assoc. Oral Maxillofac. Surg..

[32] Andrysewicz E., Mystkowska J., Dabrowski J.R., Olchowik R. (2014). Influence of self-made saliva substitutes on tribological characteristics of human enamel. Acta Bioeng. Biomech..

[33] Andrysewicz E., Mystkowska J., Kolmas J., Jałbrzykowski M., Olchowik R., Dabrowski J.R. (2012). Influence of artificial saliva compositions on tribological characteristics of Ti-6Al-4V implant alloy. Acta Bioeng. Biomech..

[34] Bansil R., Turner B.S. (2006). Mucin structure, aggregation, physiological functions and biomedical applications. Curr. Opin. Colloid Interface Sci..

[35] Mystkowska J., Łysik D., Klekotka M. (2019). Effect of saliva and mucin-based saliva substitutes on fretting processes of 316 austenitic stainless steel. Metals.

[36] Mystkowska J., Car H., Dąbrowski J.R., Romanowska J., Klekotka M., Milewska A.J. (2018). Artificial Mucin-based Saliva Preparations—Physicochemical and Tribological Properties. Oral Health Prev. Dent..

[37] Sidun J., Dąbrowski J.R. The method of fretting wear assessment with the application of 3D laser measuring microscope. Proceedings of the Advances in Intelligent Systems and Computing.

[38] Heintze S.D. (2006). How to qualify and validate wear simulation devices and methods. Dent. Mater..

[39] Hidaka O., Iwasaki M., Saito M., Morimoto T. (1999). Influence of clenching intensity on bite force balance, occlusal contact area and average bite pressure. J. Dent. Res..

[40] Hu Y., Zhong M., Bao T., Wang H. (2013). Energy dissipation in atomic-scale friction. Friction.

[41] Kim K., Geringer J. (2012). Analysis of energy dissipation in fretting corrosion experiments with materials used as hip prosthesis. Wear.

[42] Norm PN-EN ISO 10993-15:2009 (2009). Biological Evaluation of Medical Devices—Part 15: Identification and Quantification of Degradation Products from Metals and Alloys.

[43] Yakubov G.E., Macakova L., Wilson S., Windust J.H.C., Stokes J.R. (2015). Aqueous lubrication by fractionated salivary proteins: Synergistic interaction of mucin polymer brush with low molecular weight macromolecules. Tribol. Int..

[44] Barril S., Mischler S., Landolt D. (2005). Electrochemical effects on the fretting corrosion behaviour of Ti6Al4V in 0.9% sodium chloride solution. Wear.

[45] Degen T., Sadki M., Bron E., König U., Nénert G. (2014). The HighScore suite. Powder Diffr..

[46] Young R.A. (1993). The Rietveld Method.

[47] Duisabeau L., Combrade P., Forest B. (2004). Environmental effect on fretting of metallic materials for orthopaedic implants. Wear.

[48] Fouvry S., Kapsa P., Vincent L., Dang Van K. (1996). Theoretical analysis of fatigue cracking under dry friction for fretting loading conditions. Wear.

[49] Christian A., Matthias H., Anna H., Carpenter G. (2017). The mucosal pellicle—An underestimated factor in oral physiology. Arch. Oral Biol..

[50] Paszyńska E., Jurga–krokowicz J., Deręgowska–nosowicz P., Czarnecka B., Shaw H., Leszczyński R. (2006). Effect of Mouthrinses on Rheological Properties of Whole Saliva. Dent. Med. Probl..

